# The role of OCT- angiography in predicting anatomical and functional recovery after endoscopic endonasal pituitary surgery: A 1-year longitudinal study

**DOI:** 10.1371/journal.pone.0260029

**Published:** 2021-12-02

**Authors:** G. Cennamo, D. Solari, D. Montorio, M. R. Scala, L. D’Andrea, F. Tranfa, L. M. Cavallo

**Affiliations:** 1 Public Health Department, Università degli Studi di Napoli "Federico II", Naples, Italy; 2 Division of Neurosurgery, Department of Neurosciences, Reproductive Sciences and Odontostomatological Sciences, Università degli Studi di Napoli "Federico II", Naples, Italy; 3 Department of Neurosciences, Reproductive Sciences and Dentistry, Eye Clinic, University of Naples "Federico II", Naples, Italy; Roskamp Institute, UNITED STATES

## Abstract

**Purpose:**

The purpose of this study was to investigate the changes in structural spectral-domain optical coherence tomography (SD-OCT), OCT Angiography (OCTA) parameters, and visual acuity, 1 year after endoscopic endonasal approach for the removal of an intra-suprasellar pituitary adenoma compressing optic chiasm and compare outcomes with 48 hours postoperative data.

**Methods:**

Sixteen eyes of eight patients (4 males, 4 females, mean age 52 ± 11 years) were enrolled in this prospective study. The primary outcome was to evaluate the changes over time before and after surgery, analyzing the Best Corrected Visual Acuity (BCVA), Ganglion Cell Complex (GCC), Retinal Nerve Fiber Layer (RNFL) thicknesses, the retinal vessel density (VD) of Superficial Capillary Plexus (SCP), Deep Capillary Plexus (DCP), Radial Peripapillary Capillary (RPC) and the Foveal Avascular Zone (FAZ). The secondary outcome was to identify potential biomarkers that could predict visual acuity changes after 1-year follow-up.

**Results:**

When comparing SD-OCT and OCTA measurements obtained after 1 year with those observed 48 hours after surgery, GCC and RNFL were significantly improved. After a significant reduction at 48 hours, GCC thickness showed a significant increase at 1 year after surgery (p = 0.007), while a significant restoration of RNFL thickness was found at 1 year (p = 0.005), as well as the VD of SCP, DCP, and RPC values. FAZ area did not change over time. BCVA significantly improved at each time after surgery (p = 0.037, p = 0.013). A statistically significant correlation was found between the preoperative BCVA, VD of SCP, DCP, RPC, and the postoperative BCVA at 1 year (p = 0.017, p = 0.029, p = 0.031, p = 0.023).

**Conclusion:**

SD-OCT and OCTA provide helpful information to identify the retinal structural and vascular improvements 1 year after surgery. OCTA parameters could serve as potential predictive markers for visual acuity recovery at long-term follow-up.

## Introduction

Anterior visual pathway compression is a common feature of intra-suprasellar masses and may lead to visual disturbances of various entities [1]. The pressure on retinal ganglion cell (RGC) axons caused by a growing tumor can cause visual field defects, preeminently bitemporal hemianopsia, and reduced visual acuity [2]. Endoscopic endonasal surgery, allowing an optimal decompression, offers the best results in terms of surgical resection and outcomes for treating these lesions, e.g., pituitary adenomas, Rathke cleft cysts, craniopharyngiomas, accounting for up to 10–15% of all intracranial tumors, respect to other techniques, such as microsurgical one [3–5].

The acknowledgement of new methods to enlarge our understanding of morphological and functional changes in the retina and its vascular structure before and after the surgery is still needed. The reconstruction of the three-dimensional anatomical and chorioretinal vascular structures for the diagnosis of optic neuropathy has become possible using the noninvasive optical coherence tomography and, in particular, by its angiography protocol [6–12].

We recently described the predictive role of optical coherence tomography angiography (OCT-A) regarding visual recovery by investigating the early vessel density modifications at 48 hours after endoscopic endonasal pituitary surgery. Considering the data, we had assumed that early modifications in vessel density obtained by OCT-A examination might precede the OCT parameters normalization [13].

Our study aims to evaluate the changes that occur over time after pituitary adenoma removal, defining anatomical and functional retinal status with OCT and OCT -A at one-year follow-up throughout comparison of previous data.

## Materials and methods

### Population and study design

In this prospective study, we enrolled 8 patients (sixteen eyes) who underwent endoscopic endonasal approach (EEA) for the removal of intra-suprasellar pituitary adenoma compressing the optic chiasm at the Division of Neurosurgery of the University of Naples "Federico II" from January to March 2019.

Inclusion criteria were evidence of lesion compressing the chiasm at magnetic resonance imaging (MRI), the preoperative visual field (VF) defect, and the absence of previous endoscopic endonasal surgery.

Exclusion criteria were previous treatment for lesions compressing the chiasm, previous ocular surgery, congenital eye disease, high myopia (>6 diopters), diagnosis of glaucoma, any optic disc anomaly, and macula disease, low-quality OCT and OCTA images.

Each patient underwent preoperative and postoperative (48 hours and 1 year after) ophthalmological assessment, including the measurement of best-corrected visual acuity (BCVA) according to the Early Treatment of Diabetic Retinopathy Study (ETDRS) [14] (the BCVA was converted into logarithm of the Minimum Angle of Resolution (log MAR) scale for statistical calculations), slit-lamp biomicroscopy, fundus examination, structural Spectral Domain (SD)-OCT parameters (Ganglion Cell Complex and Retinal Nerve Fiber Layer) and OCTA.

The study was approved by the Institutional Review Board of the University of Naples "Federico II" (clinical trials: NCT04840771), and all investigations adhered to the tenets of the Declaration of Helsinki. Written informed consent was obtained from the patients enrolled in the study.

### Spectral-domain optical coherence tomography

SD-OCT (software RTVue XR version 2017.1.0.151, Optovue Inc., Fremont, CA, USA) was used to examine the retinal nerve fiber layer (RNFL) and ganglion cell complex thickness in all patients. The optic nerve head (ONH) analysis measured the retinal nerve fiber layer thickness, calculated along a 3.45-mm diameter circle around the optic disc. The ganglion cell complex thickness was evaluated from the internal limiting membrane to the outer boundary of the inner plexiform layer in a 7 × 7 mm grid of the macula centered 1-mm temporal to the fovea, located in the posterior pole [15]. OCTA images with a signal strength index (SSI) less than 80, and residual motion artifacts were excluded from the analysis.

### Optical coherence tomography angiography

All the subjects underwent OCTA evaluation (Optovue Angiovue System, software ReVue XR version 2017.1.0.151, Optovue Inc., Fremont, CA, USA).

The macular capillary network was evaluated in scans centered on the fovea by performing a 6 mm × 6 mm area divided, according to the ETDRS classification of diabetic retinopathy, in the whole image, fovea, and parafovea.

The AngioAnalytic^™^ software automatically analyzed the vessel density (VD) in two different retinal vascular networks: superficial capillary plexus (SCP) and deep capillary plexus (DCP) as previously described [16, 17].

The VD of the radial peripapillary capillary (RPC) plexus, analyzing the whole papillary region with an area scan of 4.5 × 4.5 mm, was automatically calculated by the Angio Vue disc mode [18].

The VD was defined as the percentage area occupied by the microvasculature in the whole scan area and all sections [17].

Angio Vue software automatically calculated the foveal avascular zone (FAZ) area in square millimeters over the 6 mm × 6 mm macular area in the full retinal plexus [19].

The 3D Projection Artifact Removal (PAR) algorithm, included in the software to remove projection artifacts, improved the quality of OCTA images.

### Statistical analysis

Statistical analysis was performed with the Statistical Package for Social Sciences (Version 25 for Windows; SPSS Inc, Chicago, Ill, USA). Normality distribution was assessed through Shapiro-wilk test. We explored VD changes over time in each retinal vascular network (SCP, DCP, and RPC), as well as structural SD-OCT parameters (GCC average and RNFL average), changes through general linear models (GLM), including age, sex as covariates, and time points as a factor of interest. Subject ID was included in all models as a random factor to account for within-subject inter-eye correlation. The multiple linear regression model was used to investigate the correlation between preoperative SD-OCT, OCTA parameters, BCVA, and postoperative BCVA. A p-value < 0.05 was considered statistically significant.

## Results

A total of sixteen eyes of eight patients (4 males, 4 females, mean age 52 ± 11 years) with optic chiasm compression signs due to an intra and suprasellar lesion were enrolled in this study. Mean follow-up after surgery was 12.5 ± 2 months.

BCVA after 48 hours and 1 year showed significant improvement compared to baseline (β = -0.014, p = 0.037; β = -0.098, p = 0.013).

After a significant reduction at 48 hours post-surgery, GCC thickness was significantly increased as compared to baseline (β = 4.571, p = 0.007), whereas RNFL thickening was observed only at 1-year follow-up (β = 6.786, p = 0.005).

After a slight increase at 48 hours, OCTA analysis revealed a significant increase in VD of SCP and DCP in each macular sector at 1 year after baseline (SCP whole: β = 4.350, p<0.001; DCP whole: β = 5.957, p = 0.023).

RPC measurements showed a significant rise in VD at 48 hours and 1 year after surgery (β = 1.950. p = 0.017; β = 4.500, p<0.001), while FAZ area did not change over time.

**Fig 1:**
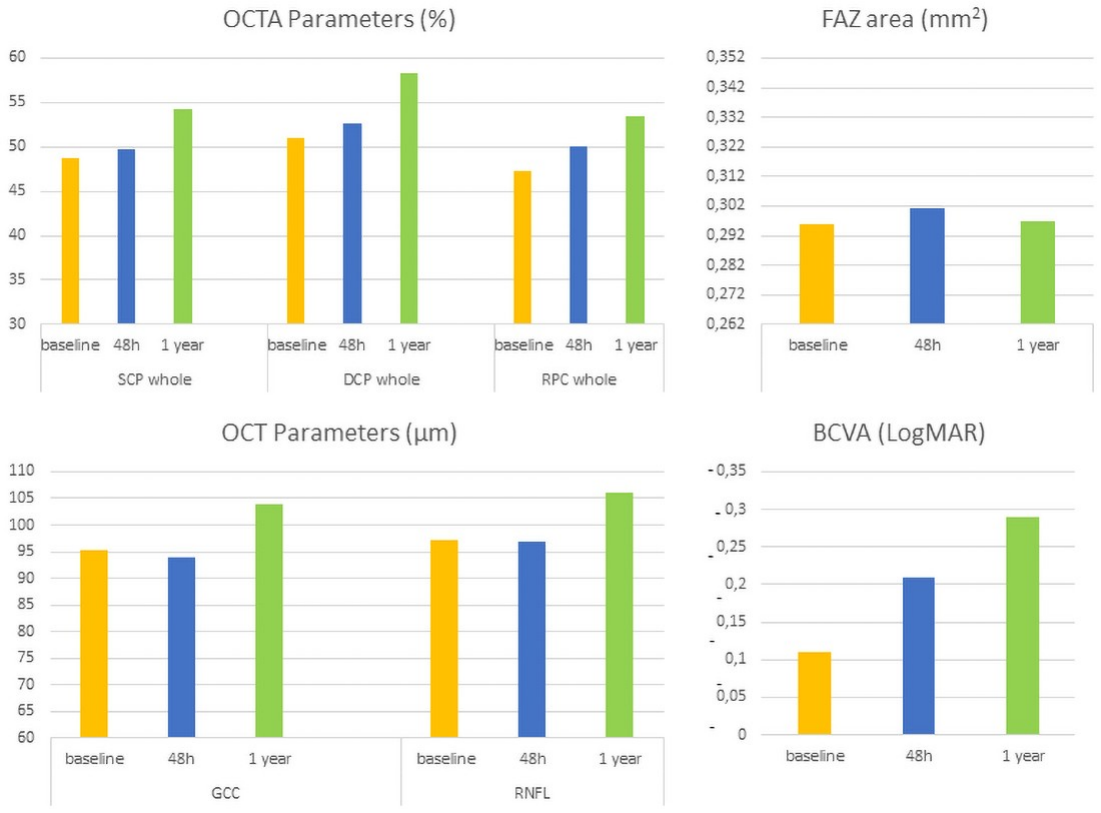
Schematic graphs of OCT and OCTA parameters measurements, and best corrected visual acuity (BCVA) at baseline, at 48 hours after surgery, and at 1-year post-surgery.

When comparing SD-OCT and OCTA measurements obtained after 1 year with those observed 48 hours after surgery, GCC and RNFL were significantly increased (β = 6.790, p = 0.017; β = 7.360, p = 0.002), as well as the VD of SCP, DCP, and RPC (Table 1, Fig 1).

**Table 1:** Differences in OCTA, SD-OCT and BCVA before and each time point after surgery via endoscopic endonasal approach.

	Baseline	48 hours	1 year	Baseline vs 48 hours		Baseline vs 1 year		48 hours vs 1 year	
				β	p-value	β	p-value	β	p-value
**OCTA parameters (%)**									
*SCP Whole*	48.81 ± 4.30	49.68 ± 3.10	54.18 ± 2.71	0.314	0.789	4.350	<0.001*	4.036	0.001*
*SCP Parafovea*	51.11 ± 7.06	51.37 ± 5.80	56.56 ± 4.71	0.621	0.756	4.371	0.029*	3.750	0.041*
*SCP Fovea*	22.83 ± 7.72	23.61 ± 5.82	28.56 ± 5.04	0.543	0.330	4.757	0.023*	6.800	0.001*
*DCP Whole*	51.01 ± 8.65	52.62 ± 7.67	58.31 ± 7.03	1.036	0.694	5.957	0.023*	6.993	0.008*
*DCP Parafovea*	56.36 ± 6.01	57.34 ± 6.94	58.68 ± 5.42	2.366	0.309	2.964	0.017*	5.300	0.021*
*DCP Fovea*	41.06 ± 9.94	43.66 ± 8.02	51.37 ± 9.10	2.893	0.304	10.121	<0.001*	7.229	0.010*
*RPC Whole*	47.22 ± 2.73	50.08 ± 2.66	53.50 ± 3.22	1.950	0.017*	4.500	<0.001*	2.500	0.002*
*FAZ area (mm* ^*2*^ *)*	0.296 ± 0.10	0.301 ± 0.14	0.297 ± 0.08	0.016	0.572	0.024	0.413	0.070	0.800
**OCT parameters (μm)**									
*GCC average*	95.31 ± 8.71	93.81 ± 7.06	103.81 ± 7.73	-2.210	0.005*	4.571	0.007*	6.790	0.017*
*RNFL average*	97.12 ± 6.15	96.87 ± 8.12	106.18 ± 9.65	-0.570	0.473	6.786	0.005*	7.360	0.002*
**BCVA (logMAR)**	-0.11 ± 0.11	-0.21 ± 0.14	-0.29 ± 0.05	-0.014	0.037*	-0.098	0.013*	-0.083	0.034*

Data expressed as mean ± standard deviation.

OCTA: Optical Coherence Tomography Angiography; SCP: Superficial Capillary Plexus; DCP: Deep Capillary Plexus; RPC: Radial Peripapillary Capillary; FAZ: foveal avascular zone; OCT: Optical Coherence Tomography; GCC: Ganglion Cell Complex; RNFL: Retinal Nerve Fiber Layer; BCVA: best corrected visual acuity.

*p<0.05.

**Fig 2:**
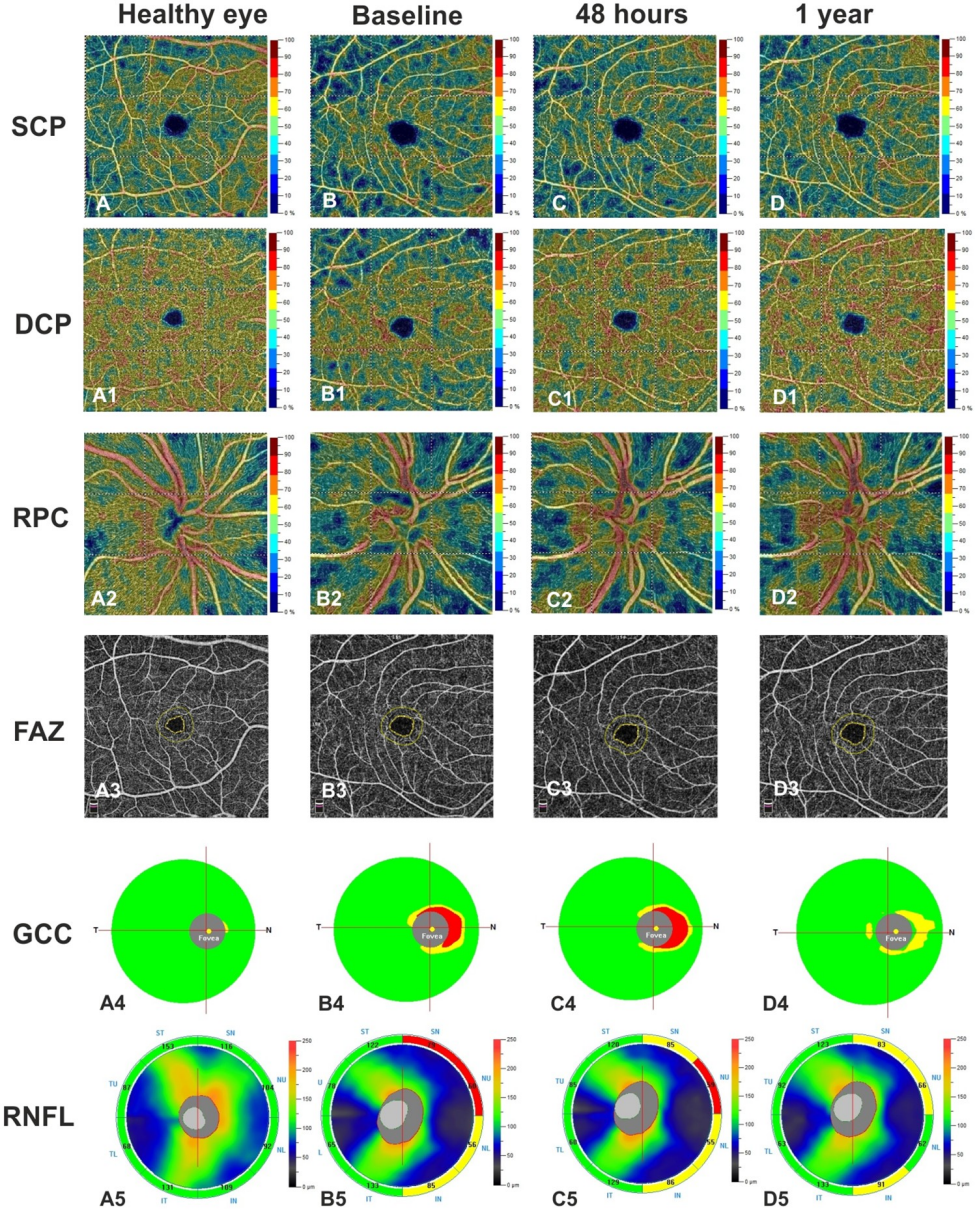
Right eye of a healthy subject (48 years-old man) shows normal vessel density of the superficial capillary plexus (SCP) (A), deep capillary plexus (DCP) (A1), radial peripapillary capillary (RPC) plexus (A2), and foveal avascular zone (FAZ) area (A3) at Optical Coherence Tomography Angiography (OCTA). Right eye of a patient (51 years-old man) with pituitary adenoma before surgery reveals a reduction of vessel density in SCP (B), DCP (B1), RPC (B2) and an increase of FAZ area (B3) compared to healthy subject. At 48 hours after endoscopic endonasal surgery, a slight increase was found in vessel density of the SCP (C), DCP (C1), while a significant increase was shown in vessel density of the RCP (C2) compared to baseline. At 1 year of follow up a significant improvement of vessel density in macular and papillary regions was found compared to other time points (D-D2). FAZ area did not change over time (B3, C3, D3). Structural Spectral Domain OCT (SD-OCT) reveals normal thickness of the Ganglion Cell Complex (GCC) (A4) and Retinal Nerve Fiber Layer (RNFL) (A5) in right eye of the healthy control. The patient shows a focal reduction in GCC and RNFL (B4, B5) at baseline, a slight decrease in GCC (C4) and an absence of changes in RNFL (C5) at 48 hours after surgery. At 1 year of follow up a significant improvement was found in both SD-OCT parameters (D4, D5).

Multiple linear regression analysis revealed a significant relationship between improved BCVA, increased GCC, RNFL thicknesses, VD of SCP, DCP, RPC at baseline, and a better BCVA 1-year post-surgery. In contrast, no significant relationship was found between GCC, RNFL, FAZ area, and BCVA (Table 2, Fig 2).

**Table 2:** Multiple linear regression model between OCTA, SD-OCT and BCVA parameters at baseline and BCVA after surgery via endoscopic endonasal approach.

	r	ANOVA	β	p value
		p value		
BCVA*-all parameters	0.762	0.012		
BCVA*-SCP whole image			-0.216	0.017
BCVA*-DCP whole image			-2.124	0.029
BCVA*-RPC whole image			-1.232	0.031
BCVA*-FAZ area			0.255	0.327
BCVA*-GCC average			-1.702	0.315
BCVA*-RNFL average			-0.924	0.229
BCVA*-BCVA			1.345	0.023

BCVA: best corrected visual acuity at baseline; BCVA*: BCVA after surgery. SCP: superficial capillary plexus; DCP: deep capillary plexus; RPC: radial peripapillary capillary plexus; FAZ: foveal avascular zone; GCC: ganglion cell complex; RNFL: retinal nerve fiber layer.

Multiple linear regression model; statistical significance P <0.05.

## Discussion

Over time, several factors or methods have been considered to expand knowledge and identify potential predictive factors for visual recovery after optic apparatus decompression, i.e., intrinsic and anatomopathological characteristics of the tumor (size, morphology, consistency, invasiveness, Knosp grade), time course prior than decompression, optic pathway diffusion tensor tractography, etc. [20]. Among clinical and ophthalmological predictive criteria, the severity of preoperative visual impairment and visual field deficits and a short period from symptom onset have been reported as favorable factors for visual restoration [21–23]. Nevertheless, none of the aforementioned factors can help the neurosurgeon to estimate the exact postoperative course in case of optic chiasm compression.

In addition, visual field alterations or BVCA assessments may be misleading because they cannot discriminate between dead or dysfunctional ganglion cells [24].

Hence, the importance of new techniques continuous development, such as the noninvasive optical coherence tomography, to expand our possibilities spectrum, as we previously reported. OCT provides valuable information in regards to the entity of compression also in patients without any visual field defects [25].

Blanch et al. [26], in their series, reported that GCC thickness detected compression disease of the chiasm before visual defects became apparent on standard automated visual field testing. Without OCT, their patients would have been labeled as having normal visual functions with no evidence of optic neuropathy.

However, the OCT-GCC parameter is reliable only for lesions with a slow growth pattern, such as pituitary adenomas, because when the damage is established abruptly, clinical evidence is frank, whereas the SD-OCT parameters can still be in a normal range [26].

In our prospective study, we explored, through a comprehensive ophthalmological examination, the changes over time of structural SD-OCT parameters (GCC average and RNFL average) and each retinal vascular network (SCP, DCP, and RPC) with the OCT-A, as well as Best Corrected Visual Acuity (BCVA), in patients that underwent trans-nasal endoscopic approach for pituitary adenomas with evidence of optic chiasm compression. Every patient was studied prospectively before, at 48 hours, and 1 year after surgery lesion removal.

Since its introduction, OCT has granted the opportunity to reconstruct the retinal morphological structure easily, while the OCTA ensured the depth-resolved visualization of all retinal vascular layers; both techniques have been applied for a wide-ranging spectrum of retinal disorders and other optic nerve diseases [27–36]. In agreement with previous studies, our preoperative values of BCVA, VD of the SCP, DCP in all macular sectors, and the VD of the RPC were significantly decreased, along with RNFL and GCC [24, 37].

As we recently described [13], the only OCT-A parameter that significantly improved in early stages after surgery was the RPC density (β = 1.950. p = 0.017), whose normalization persisted at one year of follow-up (β = 4.500, p<0.001). Accordingly, we have assumed that the initial damage triggered by chiasmal compression is due to mechanical forces that lead to lower retinal perfusion, followed by a reduced metabolic demand due to the optic injury. Consequently, we alleged that the early examination of the eventual normalization of VD could provide a prognostic factor on visual outcomes compared to the current ones such as RNFL or GCC or visual fields alteration. We decided to set to 48 hours the initial postoperative measurement to balance between the reasonable patient’s collaboration and the detection of the initial benefits of the decompression to evaluate the early retinal changes.

According to our findings, GCC and RFNL thickness were significantly increased only at 1 year of follow-up (β = 4.571, p = 0.007; β = 6.786, p = 0.005), along with a significant improvement in VD of SCP and DCP in each macular sector, confirming the sign of a successful decompression achieved.

Therefore, we can suppose that OCT parameters reveal structural changes, mirroring a condition of neuronal loss that requires more time to be restabilized.

It is consistent with other studies that described the association between RNFL and GCC thickness and the severity of visual impairment [23, 38, 39].

Conversely, OCTA assessment, evaluating the capillary flow, may allow the early detection of reduced cellular functions that may occur even before clinical symptoms, allowing a precise preoperative diagnosis. Simultaneously, it can be considered a viable instrument to assess early signs of successful blood vessels flow recovery and an accurate perspective over the functional and anatomical optic apparatus.

Finally, our multiple linear regression analysis revealed a significant relationship between improved BCVA, increased GCC, RNFL thicknesses, VD of SCP, DCP, RPC at baseline; this is in contrast with a previous paper [37], where a statistically significant correlation was noted for the OCTA parameters and not between preoperative OCT parameters (GCC and RFNL) and postoperative visual field recovery. Considering that, the resection was also made via trans-sphenoidal route, it may be partially attributed to differences in the preoperative characteristics of their patients enrolled, i.e., patients’ age, disease duration, and severity of chiasm compression, and also to the shorter period of follow-up compared to our series (three months vs 1 year), which is consistent with our theory of the longer time needed for SD-OCT parameters to normalize [2].

These findings have important implications for patient counseling regarding the expected benefits of surgery and should be incorporated into the preoperative evaluation of patients with pituitary suprasellar lesions and in their postoperative assessment for a quantifiable definition of their visual prognosis.

## Conclusions

Multidisciplinary treatment of patients is mandatory and a preoperative and postoperative exhaustive ophthalmologic assessment of the patient affected by suprasellar lesion compressing the chiasm is strictly advisable. Preoperative OCT and OCT-A devices allow noninvasive and fast vascular and anatomical retinal examination, also detecting subclinical optic alterations. Trans-nasal endoscopic surgery represents a sound and effective strategy for pituitary tumor removal. Although several factors should be taken into consideration, we retain that SD-OCT and OCTA provide helpful information to identify the retinal structural and vascular improvement after surgery. OCTA parameters, RPC in particular, could be routinely used and be claimed as a reliable predicting factor for visual recovery in those patients presenting radiological chiasm compression at onset, even without referred visual disturbances.

The limitation of this study concerns the low sample size and the absence of a control group. Longitudinal studies on larger cohorts are needed to detect the possible progression of retinal vascular alterations on long-term follow-up.
